# Zein Nanoparticles Containing Arginine-Phenylalanine-Based Surfactants: Stability, Antimicrobial and Hemolytic Activity

**DOI:** 10.3390/nano13010200

**Published:** 2023-01-02

**Authors:** Lourdes Perez, Zakaria Hafidi, Aurora Pinazo, Maria Teresa García, Manuel Martín-Pastor, Francisco Fábio Oliveira de Sousa

**Affiliations:** 1 Department of Surfactants and Nanobiotechnology, Institute for Advanced Chemistry of Catalonia (IQAC-CSIC), 08034 Barcelona, Spain; 2 Unidad de Resonancia Magnética, Área de Infraestructuras de Investigación, Universidad de Santiago de Compostela, Santiago de Compostela, 15782 A Coruña, Spain; 3 School of Pharmacy, Department of Biological & Health Sciences, Federal University of Amapa, Macapa 68903-419, Brazil

**Keywords:** arginine, phenylalanine, surfactants, zein, nanoparticles, antimicrobial, cytotoxicity, hemolysis, STD-NMR, docking

## Abstract

Although cationic surfactants have a remarkable antimicrobial activity, they present an intrinsic toxicity that discourages their usage. In this work novel zein nanoparticles loaded with arginine-phenylalanine-based surfactants are presented. The nanoparticles were loaded with two single polar head (LAM and PNHC_12_) and two with double amino acid polar head surfactants, arginine-phenylalanine (C_12_PAM, PANHC_12_). The formulations were characterized and their stability checked up to 365 days. Furthermore, the antimicrobial and hemolytic activities were investigated. Finally, NMR and molecular docking studies were carried out to elucidate the possible interaction mechanisms of surfactant-zein. The nanoparticles were obtained with satisfactory size, zeta potential and dispersibility. The surfactants containing arginine-phenylalanine residues were found to be more stable. The nanoencapsulation maintained the antimicrobial activities unaltered in comparison to the surfactants’ solutions. These results are in agreement with the NMR and docking findings, suggesting that zein interacts with the surfactants by the aromatic rings of phenylalanine. As a result, the cationic charges and part of the aliphatic chains are freely available to attack the bacteria and fungi, while not available to disrupt the cellular membranes. This approach opens new possibilities for using cationic surfactants and benefits from their extraordinary antimicrobial responses for several applications.

## 1. Introduction

Surfactants are amphiphilic molecules containing hydrophilic and lipophilic moieties, adsorbing onto interfaces and altering either the system surface or the interfacial tension. Cationic surfactants present remarkable antimicrobial properties. Despite their great bioactivity [1,2,3], they are the most irritative category to skin and mucous tissues, what limits their clinical applicability [4]. Among the multifunctional compounds, amino acid-based surfactants represent great potential. They combine an amino acid hydrophilic polar head group and an aliphatic chain as the lipophilic moiety. They have attracted much attention because of their extraordinary biodegradability and renewable sources subtracts for their synthesis.

Arginine has a flexible molecular structure and generally can be found in the zwitter ionic form, where protonation of both guanidyl and alpha amino groups as well as deprotonation of the carboxyl group are found [5]. Aside from their green profile, nowadays, these characteristics make arginine surfactants an alternative to conventional cationic surfactants for food, cosmetic and pharmaceutical industries. Amino acids with aromatic groups are also strong candidates to be part of the cationic surfactants with antimicrobial properties. Phenylalanine is one of the most studied aromatic amino acids; it contains a phenyl group connected to the amino acid moiety through a flexible methylene group. Surfactants containing phenylalanine residues have demonstrated good antimicrobial activities with optimal effectiveness at some intermediate chain length [6,7,8]. 

**Figure 1:**
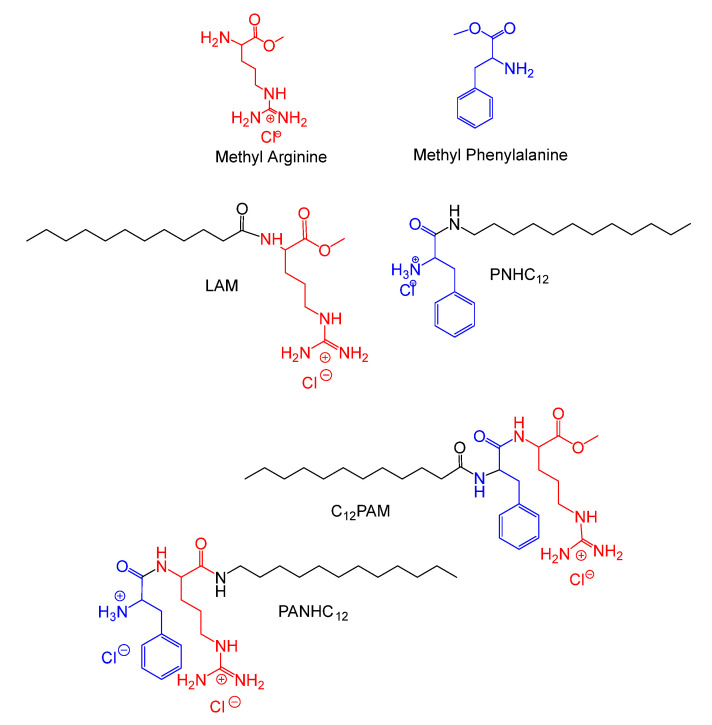
Molecular structures of amino acid-based surfactants. Lauroyl Arginine methyl ester (LAM), Phenylalanine lauroyl amide (PNHC_12_), Lauroyl Phenylalanine Arginine methyl ester (C_12_PAM), Phenylalanine Arginine lauroyl amide (PANHC_12_).

In order to improve the antimicrobial activity, new surfactants containing in the polar head both arginine and phenylalanine have recently been synthesized [3,9]. The structures and acronyms of the new surfactants are shown in Figure 1. These surfactants exhibit excellent antimicrobial, antifungal and antibiofilm activity. Notably C_12_PAM can be classified as readily biodegradable compound and also showed very good activity against numerous MRSA strains, nowadays, one of the most problematic bacteria [10].

Nanoparticles have emerged as promising antimicrobial systems due to their physicochemical and biological properties: high surface/volume ratio, prolonged bioavailability and low cytotoxicity. These aggregates often exhibit greater antimicrobial efficiency than their bulk materials [11]. Most nanoparticles described in the literature have metallic character, this causes concern owing to the fact that metals can accumulate in the body and in the environment. Then, the research for effective antimicrobials nanoparticles prepared from biodegradable and sustainable biopolymers that fulfill green chemistry principles are gaining large interest.

Corn protein zein, a prolamin found in the endosperm of maize (*Zea mays*), is a biodegradable and biocompatible biopolymer able to entrap and deliver therapeutic molecules [12,13,14,15,16,17]. Due to these interesting properties zein is an abundantly renewable resource that has long been applied in food and pharmaceutical industries [18]. However, nanoparticles prepared with pure zein have low stability, the isoelectric point of zein is close to neutral and have weak electrostatic repulsion. The incorporation of charged surfactants can effectively improve their stability. Recently, our group has demonstrated its ability to improve the antimicrobial and antibiofilm activities of therapeutic molecules [15,16,19]. Given the above background the aim of this work was to prepare and characterize a green antimicrobial system based on zein nanoparticles and cationic amino acid surfactants. The antimicrobial activity of the novel nanoparticles has been evaluated using representative microorganisms. The hemolytic activity of these nanoparticles was also evaluated to verify the possible toxicity of these systems. The synthesis of these nanoparticles using sustainable biodegradable materials such as zein and amino acid-based surfactants fulfills green chemistry principles and generates ecofriendly materials with antimicrobial activity. It should be pointed out that there have been no previous publications concerning the preparation of zein nanoparticles with amino acid-based surfactants.

## 2. Materials and Methods

### 2.1. Materials

Muller-Hinton (MH) broth and agar were obtained from Difco Laboratories (Franklin Lakes, NJ, USA). Zein, Roswell Park Memorial Institute 1640 medium (RPMI 1640), (3-(N-morpholino) propanesulfonic acid buffer (MOPS) and Dimetyl Sulfoxide (DMSO) were obtained from Sigma-Aldrich (Burlington, MA, USA). The arginine-phenylalanine based-surfactants were synthesized in our laboratory, according to the method previously described [3,9]. Their molecular structure was identified by Nuclear Magnetic Resonance ^1^H and ^13^C (Varian spectrometer at 499.803 (^1^H) and 125.233 (^13^C) MHz. and Mass-spectroscopy (Acquity UPLC system and a LCT PremierTM XE Benchtop). Their purity was checked by High Performance Liquid Chromatography with a Waters HPLC system equipped with a Kromasil 100 C8 5 µm 25 × 2.12 column. All the other reagents were analytical grade and used as received.

### 2.2. Nanoparticles’ Preparation

The nanoparticles were prepared by nanoprecipitation, according to our previous studies [15,16]. This method allows obtaining small and homogeneous droplets by increasing the surface area through the disruption in the semipolar solvent containing the polymer and the aqueous interphase. In order to access the interference of the surfactant deposition within the nanoparticles structure, two methods were proposed:

A—Zein and each surfactant were dissolved together in 10 mL of ethanol 70% *v*/*v*, followed by the addition of ultrapure water until 50 mL under constant stirring;

B—Zein was dissolved in 9 mL of ethanol 70% *v*/*v* and ultrapure water was added to the volume of 49 mL. After the nanoparticles’ formation, each surfactant dissolved in 1ml of ethanol 70% was added to the dispersion under constant stirring.

### 2.3. Nanoparticles’ Characterization

The nanoparticles were characterized in terms of size (nm), polydispersity index (pdI) and zeta potential (mV) using a dynamic light analyzer (Zetasizer^®^ Nano-ZS90, Malvern Instruments, Malvern, Worcestershire, UK). The morphology of the nanoparticles was observed using a transmission electron microscope (JEOL JEM-2010). The nanoparticles’ suspensions were cast-dried onto a 150-mesh carbon-coated copper grid. The images were taken at appropriate magnifications in order to identify and compare the characteristics of blank and surfactant loaded-zein nanoparticles.

### 2.4. Stability Evaluation

In order to determine the stability of the colloidal dispersions, the formulations were stored at room temperature (25 ± 2 °C) and refrigerator (4 ± 2 °C) and evaluated at predetermined time intervals (0, 1, 7, 30, 90 and 365 days) in terms of visual inspection, particle size, pdI and zeta potential. This analysis allows indicating the best storage condition and stability for the nanoparticles [20,21].

### 2.5. NMR Spectroscopy

NMR experiments were conducted at 25 °C on a Bruker NEO 17.6 T spectrometer (proton resonance 750 MHz), equipped with a ^1^H-^19^F/^13^C/^15^N triple resonance PA-TXI probe with deuterium lock channel and shielded PFG z-gradient. The spectrometer control software was TopSpin 4.0. The chemical shifts reported are referenced to the lock deuterium solvent. Spectra were processed and analyzed with Mestrenova v14.0 (Mestrelab Inc., Santiago de Compostela, Spain.).

In order to examine the chemical interactions between the surfactants and zein, the single compounds LAM, PNHC_12_, PANHC_12_ and C_12_PAM and binary mixtures were dissolved and homogenized in 0.6 mL of CD_3_OD:D_2_O 9:1 (*v*/*v*). The concentration of zein was fixed at 8 mM, while the mixtures were prepared at a molar ratio surfactant: zein of 50:1. NMR spectra were measured following our previous studies [22,23].

One-dimensional ^1^H spectra were measured for the individual components and the mixture surfactant–zein. The ^1^H-STD-NMR spectrum was measured for the mixture. The saturation time was set to 2 s and the STD^off^ saturation was applied at 20 ppm. The STD^on^ saturation was applied at 2.0 ppm for surfactant–zein mixture corresponding to a region of the spectrum where proton signals of zein but not surfactant are expected. The STD^on^ and STD^off^ subspectra were measured in alternate scans and subtracted by phase cycling providing the STD^off-on^ spectrum.

WaterLOGSY experiments were acquired for the individual mixtures of the surfactants LAM, PNHC_12_, PANHC_12_, C_12_PAM and zein dissolved in H_2_O:CD_3_OD 4:5 *v*/*v*. The experiments were performed with a selective 180 degrees inversion pulse applied over the water signal at 4.7 ppm by means of a gaussian shaped selective pulse of 7.5 ms covering a band-width of 118 Hz (0.15 ppm in our spectrometer). The mixing time of the experiment was set to 50 and 500 ms. Each waterLOGSY spectrum was acquired with 64 scans and a total duration of each scan of 5 s.

### 2.6. Molecular Docking Studies

**Figure 2:**
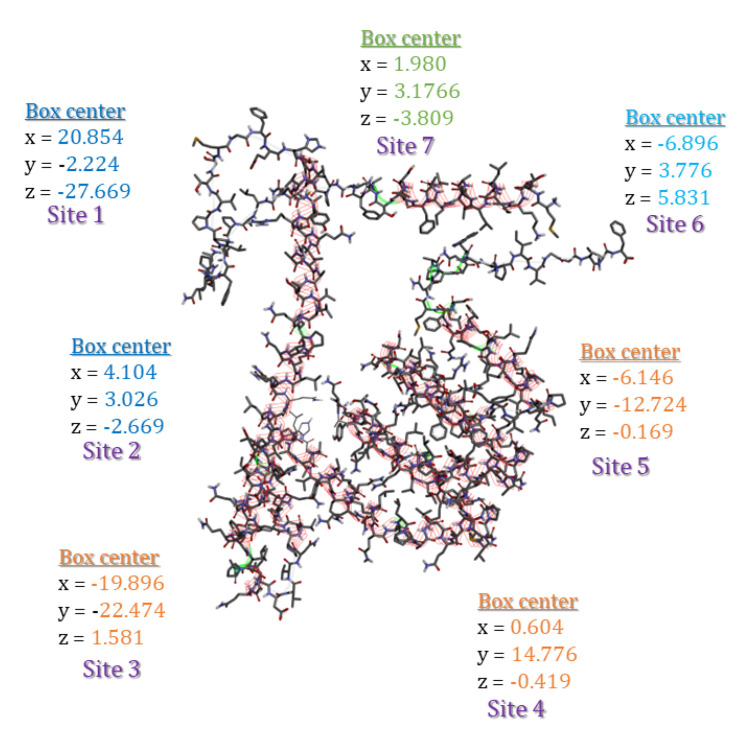
Different binding sites and their coordinates (x, y, and z) on the surface of the zein structures (Q9SYT3_MAIZE) using Discovery Studio Client v16 software.

The binding modes of the surfactants within zein protein were also investigated by molecular docking simulation using AutodockVina [24]. The structure of zein protein (Figure 2) was downloaded from the European molecular biology laboratory (EMBL-InterPro) with the code Q9SYT3_MAIZE [25]. Polar hydrogen atoms were added to the structure of the assembled protein for correcting ionization and tautomeric states of amino acid residues [26]. The putative binding sites on zein protein structure were identified using the Discovery Studio Client (version 17.2.0). All the surfactant ligands were drawn using Chemdraw12.0 software [27]. To select the most stable conformation, the geometry of these ligands was subsequently optimized using Molecular Force Field (MMFF94). The ligand and target protein files were converted to the PDBQT format to make it suitable for docking in AutoDock Vina. The interactions of complex protein-ligand conformations have been analyzed by Discovery Studio Client v16 software.

### 2.7. Minimum Inhibitory Concentration

For the antimicrobial evaluation, serial dilutions from the surfactants’ solutions and their respective nanoparticles were tested. The American Type Culture Collection (ATCC) *Bacillus subtilis* ATCC 6633, *Staphylococcus aureus* ATCC 29213, *Acinetobacter baumanniii* ATCC 19606, *Pseudomonas aeruginosa* ATCC 27853, *Staphylococcus epidermidis* ATCC 12228, *Escherichia coli* ATCC 25922, *Listeria monocytogenes* ATCC 15313 and *Enterococcus faecalis* ATCC 29212, *Candida albicans* ATCC 90028, *Candida jadinni* ATCC 60459, *Candida rugosa* ATCC 10571, *Candida glabrata* ATCC 66032, *Candida parapsilosis* ATCC 22019, *Candida tropicalis* ATCC 7349, *Candida auris* ATCC 21092 and *Candida albicans* ATCC 10231 were the bacteria and yeast used.

These microorganisms were maintained and cultured according to the specific conditions of the National Committee for Clinical Laboratory Standards [28].

The bacterial inoculum for the tests was prepared by culturing each strain in sterile Mullen Hinton (MH) broth at 37 ± 1 °C for 24 h. The yeasts were cultured in sterile RMPI 1640 buffered with 0.165M MOPS (pH 7.2) at 30 ± 2 °C. After culturing, the concentration was adjusted to 1.5 × 10^8^ colony forming units per mL (CFU/mL) for bacteria and 1.5 × 10^7^ for the yeasts (equivalent to 0.5 in the McFarland scale), which were further diluted to 10^6^ CFU/mL.

The antimicrobial activity was determined using the microdilution methodology to determine the minimum inhibitory concentration (MIC) in accordance with the CLSI guidelines [28]. Serial dilutions of the tested substances (35.6–1.125 µg/mL) were made in sterile MH broth for bacteria and buffered RPMI 1640 for yeasts in 96-well sterile microplates and incubated together with the inoculum. Blank zein nanoparticles in the same concentration of the loaded nanoparticles and sterile saline solution were used as negative controls, while chlorhexidine gluconate at 2% (CHX) and Amphotericin B at 2 μg/mL (AMB) were used as positive controls for bacteria and yeasts, respectively. MIC was defined as the lowest concentration able to inhibit the inoculum growth. All assays were performed in triplicate under strictly aseptic conditions.

### 2.8. Hemolytic Activity

Rabbit blood was supplied by the Animal Experimentation Unit (Instituto de Química Avanzada de Cataluña—IQAC). Erythrocytes were separated from heparinized blood, before preparing their suspension for the hemolysis test [29]. In this process, blood was centrifuged at 3000 rpm for 10 min. After discarding the supernatant, the erythrocytes were suspended in phosphate buffer isotonic saline solution (PBS 7.4), to remove white cells and other debris followed by centrifugation at 3000 rpm for 10 min. This washing step was repeated three times. An erythrocyte stock suspension was diluted in PBS to 8 × 10^9^ cells/mL, equivalent to an optical density of 1.8–2.1 at λ = 540 nm after total hemolysis (control, 100% hemolysis caused by ultrapure water).

A fixed amount of each testing sample (500 µL) was placed in polystyrene microtubes. An aliquot of 25 μL of erythrocyte suspension was added to each microtube and the volume was completed to 1 mL with PBS pH 7.4. The mixture was gently shaken at room temperature for 10 min. Finally, the microtubes were centrifugated (10,000 rpm) at room temperature for 5 min. Thereafter, the supernatant was carefully extracted and its absorbance was measured spectrophotometrically at λ = 540 nm. The percentage of hemolysis was calculated by comparing the absorbance of the sample (supernatant) with that of the control samples which were totally hemolyzed using ultrapure water.

Before the test, the basal hemolytic activity (0%) was checked using PBS 7.4 as control, while the 100% hemolysis was determined using ultrapure water. The last was used to determine comparatively the individual hemolytic activity (in %). After that, blank and surfactant-loaded zein nanoparticles and their solutions were assayed using the adjusted erythrocytes suspension.

To quantify the leached hemoglobin, the absorbance of the supernatant was measured at λ = 540 nm using PBS 7.4 as blank, according to the following equation:

$$\%\;haemolysis=\frac{Abs_{test\;compound}}{Abs_{Basal\;haemolytic\;activity}}$$

### 2.9. Statistical Analysis

Data were expressed as means ± standard deviation, submitted to one-way analysis of variance (ANOVA) test followed by Tukey’s multiple comparison. The statistical significance for all tests was set at *p* < 0.05, using the GraphPad Prism 5.0 software.

## 3. Results and Discussion

### 3.1. Nanoparticles Characterization

The arginine-phenylalanine-based surfactants and zein resulted in a viable association, both chemically, functionally and environmentally friendly, as both can be obtained from renewable sources.

**Figure 3:**
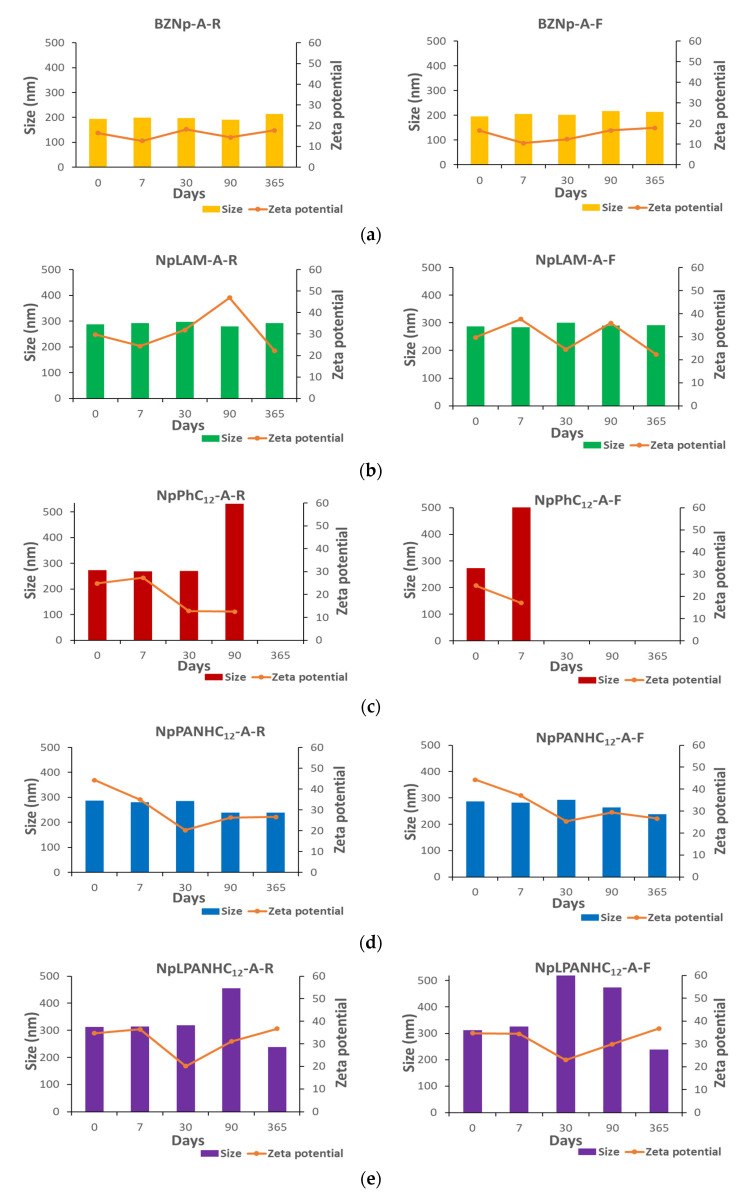
Size (nm) and zeta potential (mV) of: (**a**) blank, (**b**) LAM, (**c**) PNHC_12_, (**d**) PANHC_12_ and (**e**) C_12_PAM loaded-zein nanoparticles prepared by Method A and stored at room temperature (R) and freezer (F) over 365 days.

**Figure 4:**
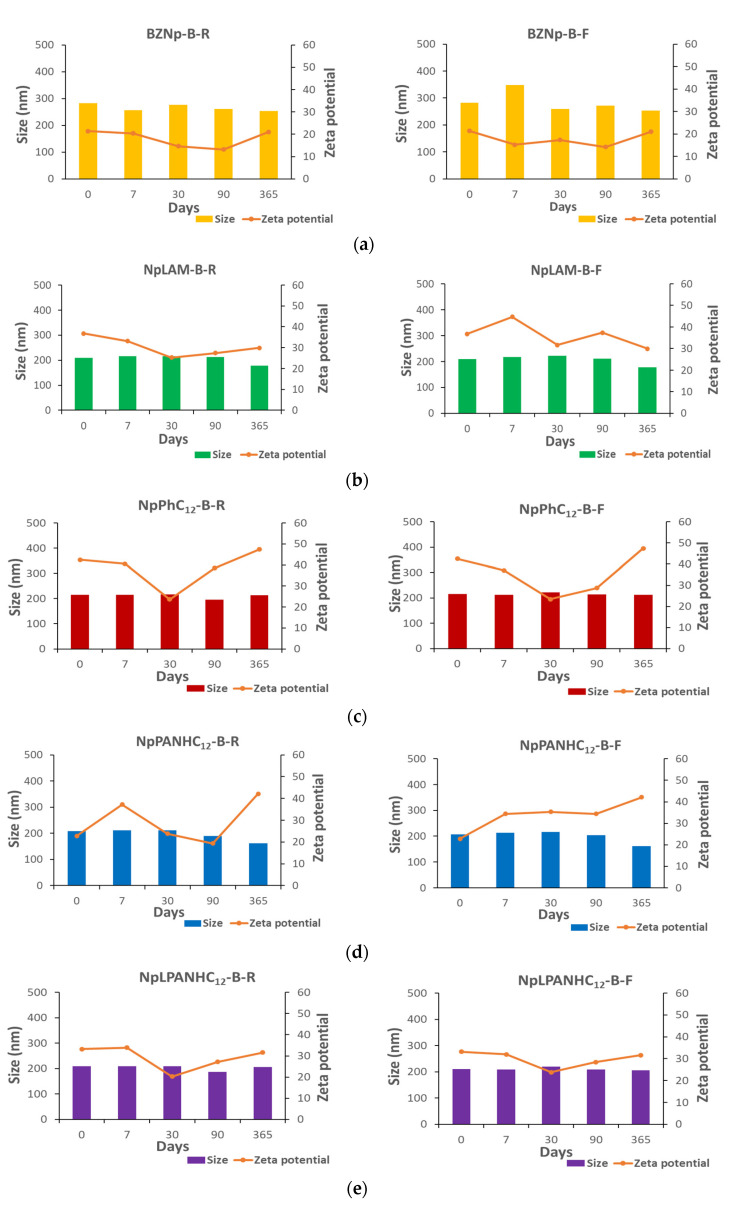
Size (nm) and zeta potential (mV) of: (**a**) blank, (**b**) LAM, (**c**) PNHC_12_, (**d**) PANHC_12_ and (**e**) C_12_PAM loaded-zein nanoparticles prepared by Method B and stored at room temperature (R) and freezer (F) over 365 days.

The nanoparticles stored at room temperature (25 °C) and freezer (4 °C) were characterized in terms of size, pdI and zeta potential immediately after their preparation and at pre-set times over 365 days (Figure 3 and Figure 4). During the stability evaluation, the formulations in which a substantial disruption and/or precipitation occurred were immediately removed from the assay.

Blank zein nanoparticles (BZNp) prepared by methods A and B were found monodispersed: 194.3 and 282.1 nm and pdI of 0.100 and 0.051, respectively. The formulation B was found slightly bigger than that prepared by method A. The only difference between those methods is the order of addition of the solutions. Therefore, the nanoparticles are conditioned by the preparation method used. The blank zein nanoparticles presented positive zeta potential (>+15 mV).

LAM-loaded zein nanoparticles (NpLAM) presented a remarkable difference in size when prepared by method A (NpLAM-A, 287.0 nm) and by method B (NpLAM-B, 208.8 nm) (Figure 3 and Figure 4), unlike the blank nanoparticles. They were found to be homogeneous: pdI 0.048 and 0.057 and presented a zeta potential over +30 mV, indicating a great level of stability. The larger nanoparticles obtained in method A could be due the incorporation of the surfactant in the matrix of the nanoparticles, as it has been incorporated in the zein solution previously to the nanoparticles’ formation, occupying more extensively the protein pockets. Contrariwise, the surfactant could have been disposed majorly in the surface when method B was used. With method B repulsions between the cationic charges are produced, preventing aggregation and particle size growth and possibly improving the polymer packing and nanoparticles dispersion, reducing as a result their size, even when compared to the blank formulation. Nonetheless, the LAM-loaded nanoparticles remained mostly stable in terms of size and zeta potential over 365 days (Figure 3 and Figure 4).

The nanoparticles containing PNHC_12_ presented an unusual behavior compared to their homologues. While the nanoparticles prepared by method B (Np PNHC_12_—B), i.e., superficial loading in the pre-formed nanoparticles, was found stable over the studied period, those prepared by method A (Np PNHC_12_—A) disrupted, after 90 and 7 days, when stored at room temperature and refrigerator, respectively. In view of that, a reasonable hypothesis is that the bulky aromatic residue of the phenylalanine found in PNHC_12_ molecule could hinder over too many zein intramolecular interactions in the A preparation, causing its precipitation. This fact supports the difference in the surfactant incorporation within zein pockets according to the preparation procedure. The zeta potential followed this pattern, remaining over +30 mV on the formulation prepared by method B and dropping down in the formulation prepared by method A.

Unlike the previous surfactants, PANHC_12_ and C_12_PAM nanoparticles remained very uniform along one year storage (Figure 3 and Figure 4). The arginine-phenylalanine nanoparticles may have hindered steric spaces within zein molecular structure [2] PANHC_12_ formed the foremost stable nanoparticles among the surfactants. NpC_12_PAM increased slightly their size during the storage period. The nanoparticles prepared by method A were found to be larger than those prepared by method B for both surfactants, which could be an indicative of the zein pockets occupation by the surfactants, promoted by the different preparation method (Figure 3 and Figure 4). Additionally, NpC_12_PAM prepared by method A were found to be larger than NpPANHC_12_, which indicates that the position of phenylalanine residue in the surfactant molecule also hinders important differences in the steric and conformation of zein nanoparticles formed (Figure 3 and Figure 4). Except for NpC_12_PAM stored at room temperature, all the other nanoparticles kept a zeta potential above +30 mV (Figure 3 and Figure 4).

### 3.2. TEM Morphological Appreciation

**Figure 5:**
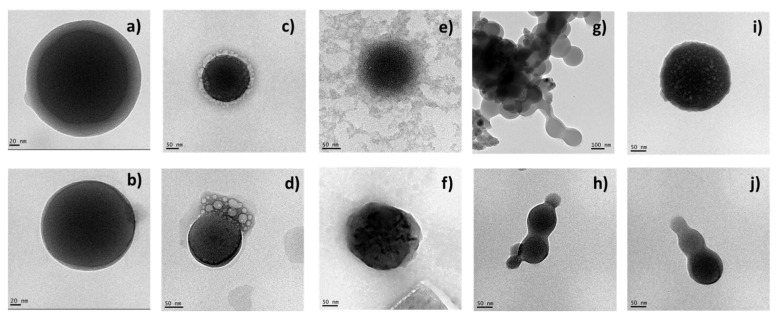
TEM images of: blank nanoparticles (**a**,**b**), LAM (**c**,**d**), PNHC_12_ (**e**,**f**), PANHC_12_ (**g**,**h**) and C_12_ PAM (**i**,**j**) loaded-zein nanoparticles prepared by methods A (first line) and B (second line).

The blank and surfactant-loaded zein nanoparticles were observed by transmission electronic microscopy (TEM). The images obtained allowed identifying the morphology and confirm the size results of the nanoparticles obtained by DLS technique (Figure 5).

Most nanoparticles were found monodisperse, spherical and uniform (Figure 5). LAM (Figure 5c) and PNHC_12_ (Figure 5e) nanoparticles prepared by method A presented a core (have a feeble band) on their surface, suggesting the presence of surfactants. Some micelles-like structures were found over LAM nanoparticles prepared by method B (Figure 5d). PANHC_12_ (Figure 5g,h) nanoparticles and C_12_PAM (Figure 5j) prepared by method B formed aggregates when dried. Though, those could be easily dispersed when remaining in the vehicle, such as measured by size and pdI measurements. The diameters measured in TEM images are in agreement with the DLS measurements (Figure 3 and Figure 4). According to the results of TEM analysis and zetasizer measurements, it is possible to confirm a good stability for zein nanoparticles containing the surfactants tested, despite the preparation method used.

### 3.3. NMR Studies

STD experiment provides easy access to the ligand binding epitope and affinity. The water-LOGSY experiment relies on the transference of NOE from the water resonance. In a sample containing a small molecule and a macromolecular receptor, the evidence in the spectrum of signals of the small molecule with the same phase as the water peak indicates that there is weak to intermediate binding affinity between the two molecules and the water found at the macromolecule binding pockets [30]. Those experiments were used to evaluate the surfactant: zein mechanism of interaction and the role of the water co-solvent in the, such as occurs in the nanoparticles´ formation

**Figure 6:**
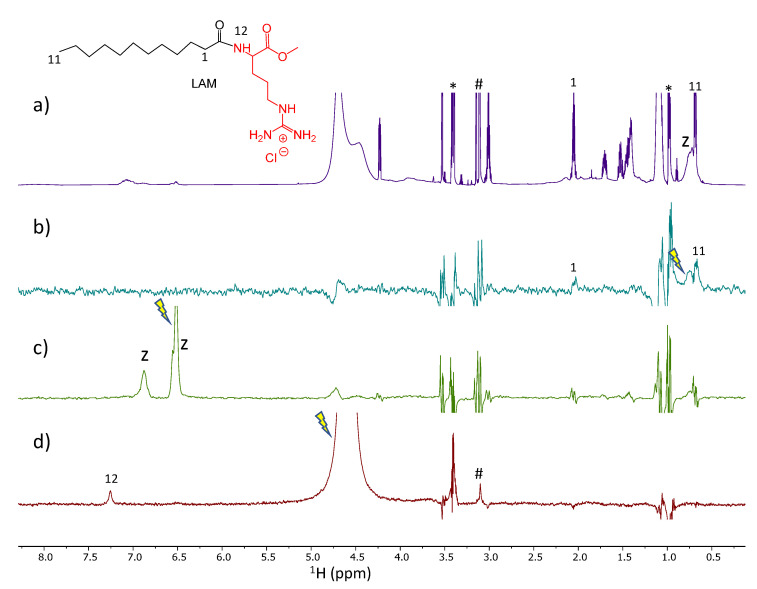
Spectra of LAM and zein mixture. (**a**) ^1^H NMR, (**b**,**c**) STD^off-on^ spectra with saturation at 0.82 and 6.6 ppm, respectively and (**d**) WaterLOGSY (mix. 500 ms). The arrow indicates the signal that is selectively on-saturated in STD spectra or selectively inverted in waterLOGSY. The assignment is provided for some relevant protons giving STD or waterLOGSY responses. The z refers to zein peaks, the asterisk denotes protons of ethanol impurity and the ampersand denotes an unidentified impurity.

**Figure 7:**
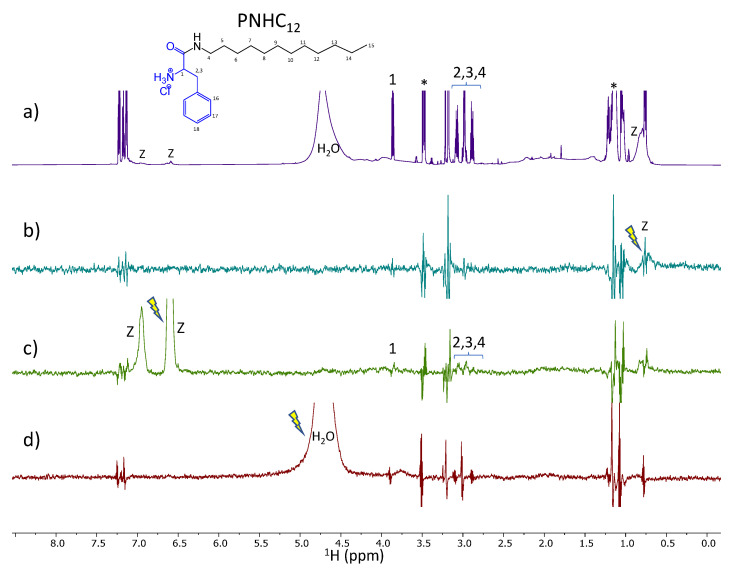
Spectra of PNHC_12_ and zein mixture. (**a**) ^1^H NMR, (**b**,**c**) STD^off-on^ spectra with saturation at 0.82 and 6.6 ppm, respectively and (**d**) WaterLOGSY (mix. 500 ms). The arrow indicates the signal that is selectively on-saturated in STD spectra or selectively inverted in WaterLOGSY. The assignment is provided for some relevant protons giving STD or waterLOGSY responses. The z refers to zein peaks and the asterisk denotes protons of ethanol impurity.

**Figure 8:**
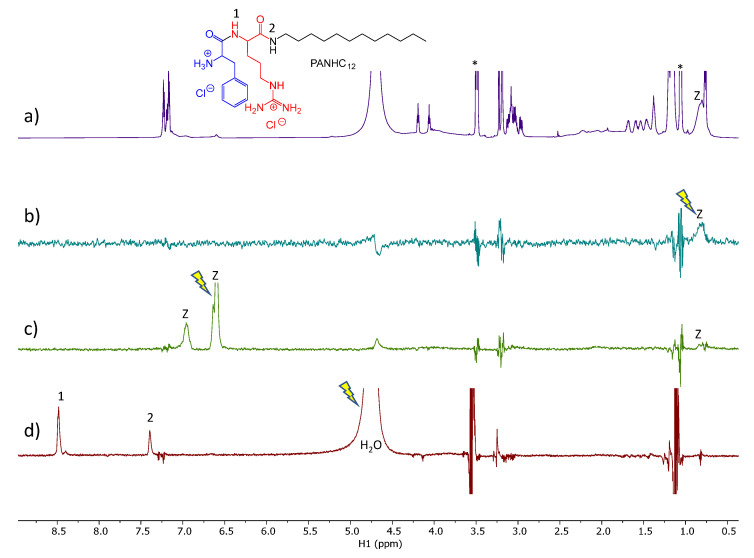
Spectra of PANHC_12_ and zein mixture. (**a**) ^1^H NMR, (**b**,**c**) STD^off-on^ spectra with saturation at 0.82 and 6.6 ppm, respectively and (**d**) WaterLOGSY (mix. 500 ms). The arrow indicates the signal that is selectively on-saturated in STD spectra or selectively inverted in WaterLOGSY. The assignment is provided for some relevant protons giving STD or WaterLOGSY responses. The z refers to zein peaks and the asterisk denotes protons of ethanol impurity.

**Figure 9:**
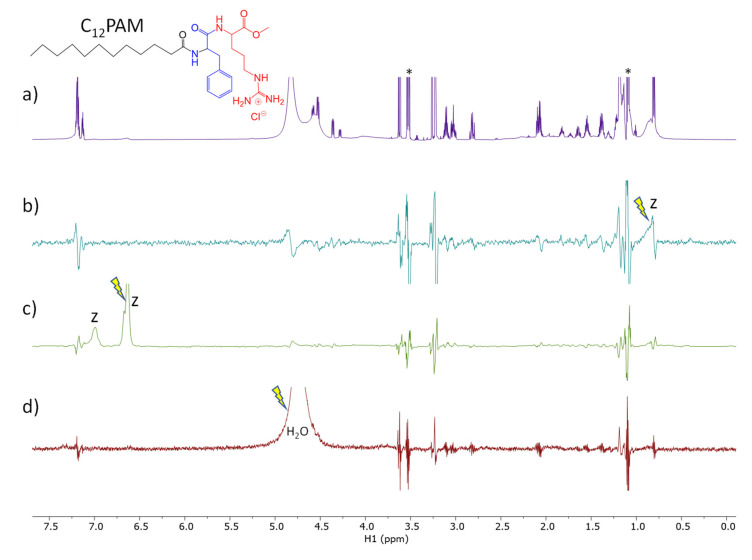
Spectra of C_12_PAN and zein mixture. (**a**) ^1^H NMR, (**b**,**c**) STD^off-on^ spectra with saturation at 0.82 and 6.6 ppm, respectively and (**d**) WaterLOGSY (mix. 500 ms). The arrow indicates the signal that is selectively on-saturated in STD spectra or selectively inverted in WaterLOGSY. The assignment is provided for some relevant protons giving STD or waterLOGSY responses. The z refers to zein peaks and the asterisk denotes protons of ethanol impurity.

The spectra obtained from the STD-^1^H NMR spectroscopy for surfactants–zein samples are displayed in Figure 6, Figure 7, Figure 8 and Figure 9. LAM and PNHC_12_ are surfactants with just one amino acid and the arginine-phenylalanine surfactants (PANHC_12_ and C_12_PAM) contain two amino acids on the polar head in which the difference remains solely in the position of the phenylalanine group. Therefore, their ^1^H NMR spectra are very alike, varying solely the position of some peaks. For surfactant-zein mixtures, a signal in the STD^off-on^ spectrum upon saturation of zein signals evidences the formation of a transient complex between the surfactant and zein. In the study of Araujo et al., 2021 [15], anacardic acid, a 12C phytochemical with lipidic structure, was found to associate with zein in two forms involving the aromatic and aliphatic moieties. The formation of complexes between PNHC_12_, PANHC_12_ and C_12_PAM-zein could explain the reduction in their hemolytic activity when encapsulated in zein nanoparticles. As the aliphatic moieties were complexed to the protein, the cationic moieties (arginine groups) were still found available to interact biologically with the bacterial and fungi, maintaining their biocide activity mostly equivalent to their solutions. Nonetheless, PNHC_12_, which has a sole aliphatic chain was found to lose its antibacterial activity when formulated in the inner structure of the nanoparticles (Method A).

The STD spectra of LAM and zein mixture (Figure 6b) with on-saturation in the region of the aliphatic protons of zein shows the responses of two aliphatic protons of LAM (protons H-1 and H-11) probing their transient binding interaction of these two apolar moieties. The STD spectrum with on-saturation of the aromatic protons of zein (Figure 6c) does not show any response of LAM. The waterLOGSY spectrum shows the response of the amide proton of LAM as a result of chemical exchange with the H_2_O protons (Figure 6d). Overall, it indicates that the polar amide group is well exposed to bulk water.

The STD spectra of PNHC_12_ and zein mixture (Figure 7b) with on-saturation in the region of the aliphatic protons of zein does not show any response. In contrast, the STD spectrum with on-saturation of the aromatic protons of zein shows responses of several protons of PNHC_12_ (protons 1 to 4 in Figure 7c) probing a more robust binding interaction of the aromatic surfactant. The waterLOGSY spectrum (Figure 7d) does not show any response possibly reflecting that PNHC_12_ has no access to the external water by its entrapment to zein structure upon the nanoparticles’ formation.

The STD spectra of PANHC_12_ and zein mixture of Figure 8b,c with on-saturation of the aliphatic and aromatic protons of zein, respectively, does not show any response of PANHC_12_. The waterLOGSY spectrum shows the response of the two amide protons of PANHC_12_ as result of chemical exchange with the H_2_O protons. Overall, it indicates that the polar amide groups are well exposed to bulk water.

The STD spectra of C_12_PAM and zein mixture of Figure 9b,c with on-saturation of the aliphatic and aromatic and protons of zein, respectively, also does not show any response of C_12_PAM. Additionally, the waterLOGSY spectrum does not show any response (Figure 9d), possibly reflecting that PANHC_12_ has no access to the external water due to its inner entrapment to zein.

### 3.4. Molecular Docking

**Figure 10:**
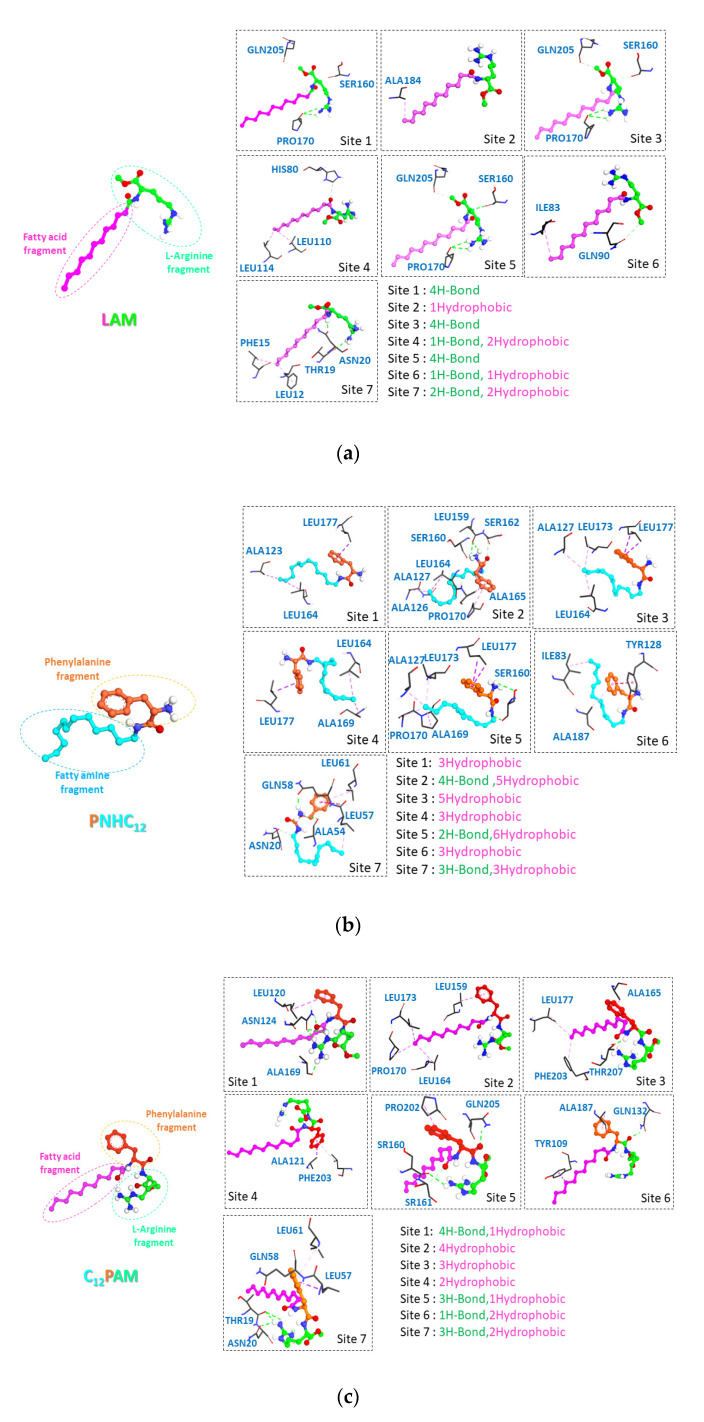

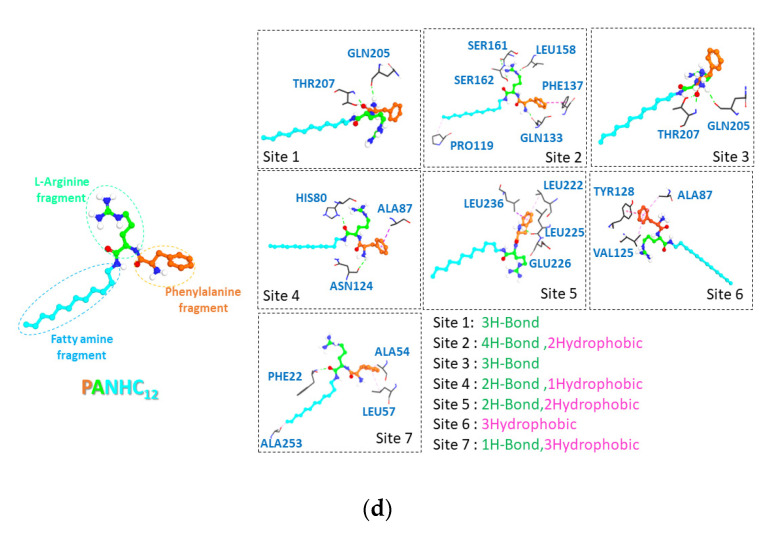
Different types of interaction on the surface of the zein structures (Q9SYT3_MAIZE) in different binding sites for (**a**) LAM, (**b**) PNHC_12_, (**c**) C_12_PAM and (**d**) PANHC_12_.

Molecular docking was considered a powerful computational method capable of predicting the binding affinity of new molecules in the site of the target receptor binding modes, as well as characterizing the different connection modes. In order to understand how zein-surfactants nanoparticles are brought into contact with each other, molecular docking simulations were performed. The surfactants showed a good affinity to zein protein pockets as shown in Tables S1–S4 (Supporting Information). All the compounds exhibited strong free energy binding between −6 and −9 kcal/mol. All studied ligand analogs showed good promising results interactions (3D) with different amino acid residues and the results are shown in Figure 10.

The interaction mode of the surfactants to the different active sites of the zein protein, allowed us to understand in an approximate way and also to predict hypotheses on how and why the antimicrobial activity of certain surfactants changes vis à vis their environment (in solution and nanoencapsulated).

According to the antimicrobial results, the surfactants maintained the antibacterial activity, except for Np PNHC_12_. In the interaction modes of PNHC_12_, a predominance of hydrophobic type interactions was noticed, in which the participation of the hydrophobic alkyl chain was always presented in the seven possible modes of interactions (see Table S2). This aspect agrees with the NMR findings presented above (Figure 7). In contrast, the participation of the cationic polar head in the interactions was not always observed (just in three sites), which gives an idea that the positive charge N^+^ of NpPNHC_12_ has a high probability of being free which allows the PNHC_12_ to keep a certain activity against microorganisms in nanoparticles forms. Although NpPNHC_12_ had no evident activity, this can probably come down to the effectiveness of NpPNHC_12_ at the level of the bacterial walls. The availability of the cationic charge initially allows the existence of electrostatic interaction between N^+^ and the negative charged of cell walls of microorganisms, but to finish their modes of action the surfactant molecules always need to carry out a hydrophobic interaction into bacterial lipid membrane [31], and this cannot be achieved in the case of NpPNHC_12_ because of the unavailability of the hydrophobic part, which always remains in interaction with the residues of zein. This has been well determined in different studies indicating that the low hydrophobicity character of surfactants decreases their antimicrobial activity [32].

Alternatively, the mode of interaction of LAM indicated that most interactions found in the different active sites of the protein are H-bonds in which the N^+^H_2_ of the guanidine function was implicated against different residues; which indicates that the cationic part of LAM is not always available in the nanoparticle environment which normally affects the antimicrobial activity of LAM nanoparticles, but on the contrary LAM showed immutability against microorganisms in both solution and nanoencapsulated, this comes from the canonical forms of guanidine (Figure S1), as the positive charge remains permanent on the two nitrogen’s of the guanidine function, this allows the LAM molecules to retain their positive charge even when one of the nitrogen’s has engaged in hydrogen interaction with some residues of zein. The same aspect was also observed in the mode of interaction of C_12_PAM, where just 3 sites out of 7 had the alkyl chain involved in the hydrophobic interactions with different residues. In the other sites we notice a predominance of H-Bond interactions where different fragments of the molecules are engaged, sometimes (in three sites) the guanidine function participates in the interaction, although it does not affect the guanidine charger because of its canonical forms. This shows that the main parts of the molecules (hydrophobic and polar) are always available in the nanoparticles, what explains the reduced antibacterial activity of this molecule in the solution and also in the nano environment.

For PANHC_12_ molecule with two positive charges, there were only two cases where the alkyl chain participated in hydrophobic interaction (pocket 2 and 7). In contrast, in the seven active sites of zein, we noticed a predominance of H-Bond interactions where different fragments of the molecules are engaged, among these cases the positive charge N^+^H_3_ and the guanidine function were simultaneously interaction in just one case. In other cases, a permanent participation of the positive charges was found, where N^+^H_3_ or guanidine participate in the interaction, which indicates that at each time one of the positive charges is absolutely available together with the hydrophobic part, which explains the non-changeability of the biological activity of PANHC_12_ with respect to the solution and its nanoparticles.

### 3.5. Antimicrobial Activity

The development of new and effective therapeutic systems is necessary for combating microbial resistance. Cationic surfactants have become potential candidates to overcome microbial resistance; besides their noteworthy antimicrobial activity, their mechanism of action limits the developed of resistance in sensible microorganisms [7,33]. Nanoparticles prepared with renewable and sustainable compounds such as zein and cationic amino acid-based surfactants can be a promising approach to solve this problem.

**Figure 11:**
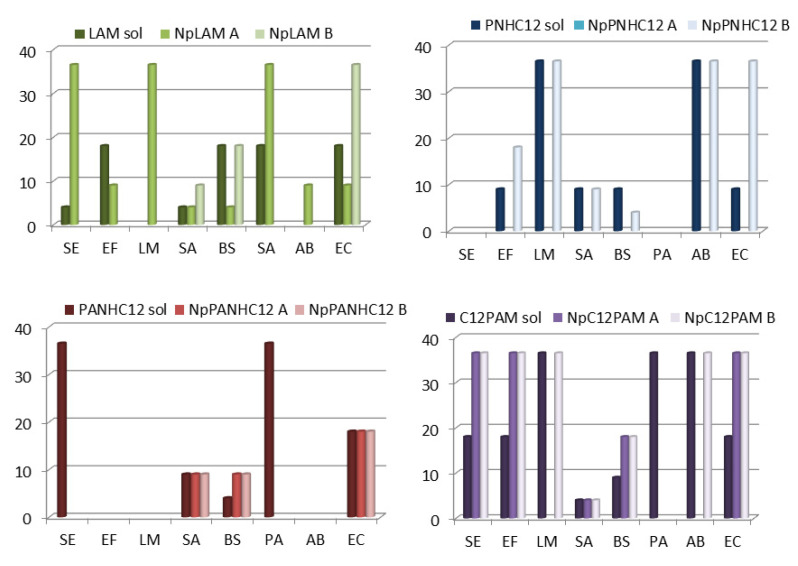
MIC values (µg/mL) for LAM, PNHC_12_, PANHC_12_ and C_12_PAM solutions and loaded-zein nanoparticles prepared by methods A and B against the following bacteria: SE—*Staphylococcus epidermidis*, EF—*Enterococcus faecalis*, LM—*Listeria monocytogenes*, SA—*Staphylococcus aureus*, BS—*Bacilus subtilis*, PA—*Pseudomonas aeruginosa*, AB—*Acinetobacter baumannii*, EC—*Escherichia coli*. Negative and positive controls were sterile MH broth and chlorhexidine gluconate at 2% (CHX), for whose growing and non-growing bacteria were observed. BZNp did not present any inhibitory activity as well.

**Figure 12:**
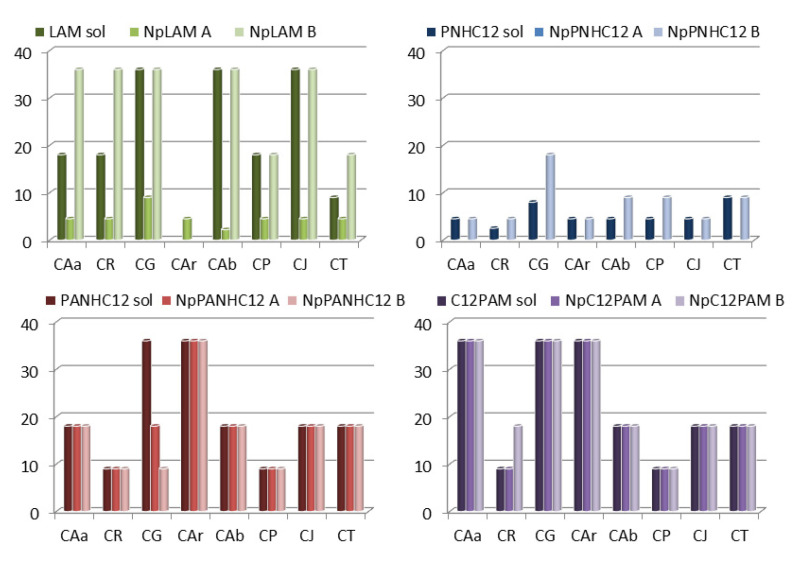
Determination of the MIC (µg/mL) for LAM, PNHC_12_, PANHC_12_ and C_12_PAM solutions and loaded-zein nanoparticles prepared by methods A and B against yeasts: CAa—*Candida albicans*, CR—*Candida rugosa*, CG—*Candida glabrata*, CAr—*Candida auris*, Cab—*Candida albicans*, CP—*Candida parapsilosis*, CJ—*Candida jadinni*, CT—*Candida tropicalis*. Negative and positive controls were sterile RPMI 1640 medium and Amphotericin B at 2 μg/mL (AMB), for whose growing and non-growing were observed. BZNp did not present any inhibitory activity as well.

The antimicrobial activity of the new nanoparticles prepared in this work was determined using representative bacteria and yeast. Figure 11 and Figure 12 show the MIC for blank zein nanoparticles (BNp-A and B) and the 4-surfactant loaded-zein nanoparticles prepared by methods A and B (NpLAM-A and B, NpPNHC_12_-A and B, NpPANHC_12_-A and B, C_12_PAM-A and B). The MIC values expressed in these figures correspond to the concentration of cationic surfactant in the nanoparticles, while the same concentration and vehicle was used in the surfactant solution.

Regarding the antimicrobial responses, a greater activity was found against yeasts than bacteria (Figure 11 and Figure 12). In overall, the surfactants maintained the antibacterial activity, except for NpPNHC_12_ prepared by method A, which lost completely its activity (Figure 11), unlike its solution and the nanoformulation prepared by method B, whose presented a good activity.

Regarding the preparation method, NpLAM-A were found more active than those prepared by method B. In contrast, NpPANHC_12_ and NpC_12_PAM presented equal activity (Figure 11 and Figure 12), despite the preparation method used. Moreover, few cases of low/lack activity of the surfactant-nanoparticles were observed.

Lauroyl arginine methyl ester (LAM) is a biodegradable cationic surfactant that exhibits good antimicrobial activity against different bacteria and fungus and, interestingly, can be classified as a readily biodegradable compound [7]. This surfactant exhibited activity at the tested concentrations against 6 out of 8 bacteria assayed (Figure 11). Notice that in this work the highest concentration tested was 36.5 µg/mL. Moreover, LAM nanoparticles prepared with method A (NpLAM-A) presented very low MIC values and were active over all the bacteria tested (Figure 11), and in some cases exhibited better antimicrobial activity than the free surfactant. In fact, at 36.5 µg/mL, these nanoparticles were active against *L. monocytogenes* and *A. baumannii*, two microorganisms that nowadays cause important health problems. When method B was used (Np-LAM-B) the nanoparticles showed lower antimicrobial activity than LAM; in some of the tested bacteria these nanoparticles were not effective (Figure 11), indicating a clear difference in the activity promoted by the incorporation method used.

The phenylalanine derivative PNHC_12_ also exhibited good antibacterial activity against 6 of the 8 bacteria studied. These results agree with those reported by Joondan et al., 2014 [8]. The preparation method used to obtain the zein nanoparticles with these compounds affected seriously their antimicrobial activity. Nanoparticles prepared by method B kept the antibacterial potency of the surfactant solution, unlike the method A preparation, which lost completely its antibacterial activity when the surfactant was nanoencapsulated together with zein solution during its preparation. This important difference can be ascribed to their different size and binding mode, such as aforementioned (Figure 7 and Figure 10). NpPNHC_12__A had higher size and lower stability than NpPNHC_12__B. It was reported that the smaller particle size and improved stability are general parameters that correlate with the antimicrobial efficiency. Smaller nanoparticles have large specific surface areas which result in higher probability to interact with the bacterial membrane [11]. Moreover, PNHC_12_ was found to interact more extensively with zein and as a result it may have hindered its activity when loaded in the inner structure.

PANHC_12_ was found to be the least active against bacteria among the surfactants tested. For instance, only 5 strains were sensible to its solution and 3 to the nanoparticles, despite the preparation method used (Figure 11).

C_12_PAM exhibited the broadest activity spectrum inhibiting all Gram-positive and Gram-negative strains tested. Recent studies showed that this surfactant had very good activity against bacteria and fungus. It exhibited good antibiofilm activity against several resistant candida strains and, interestingly, it was found to be a very good biodegradable compound [3,9]. The nanoencapsulation did not interfere significantly with its activity, those prepared by method-A (NpC_12_PAM-A) and method B (NpC_12_PAM-B) were active over 6 and 7 strains, respectively, at MIC values equal or below 36.5 μg/mL (Figure 11). The only species resistant to both nanoparticles at the highest concentration tested was *P. aeruginosa* (Figure 11).

The nanoparticles as well as the cationic surfactants used to formulate them, showed higher efficiency against Gram-positive microorganisms. It was found that the mechanism of the C_12_PAM against bacteria mainly involve electrostatic interactions between the positive charge of this compound and the negatively charged bacterial cellular membranes followed by the permeation of the alkyl chains into the intermembrane region, giving rise to a leakage of cytoplasmatic material [3,9]. According to the literature, one of the main processes underlying the antibacterial effects of nanoparticles is also the disruption of the bacterial cell membranes. This mechanism of action explains the greater efficiency showed by bulk solution and the nanoparticles against Gram-positive than Gram-negative bacteria. The former contains a single phospholipid cellular membrane and a thicker cell wall composed of peptidoglycan, whereas the latter are encapsulated by two cellular membranes and a rather thin peptidoglycan cell wall. Interestingly, this mode of action allows these surfactants to disturb a broad spectrum of microorganisms and reduce the probability of bacterial drug resistance because bacterial membrane is difficult to change through only a few genetic mutations.

Nowadays, antibacterial nanoparticles have emerged as promising systems. Nanoparticles composed of biodegradable polymers such as chitosan have been extensively studied to increase the stability, poor solubility and antimicrobial efficiency of essential oils [34]. Self-assembly nanoencapsulation technology based on the zein protein was also employed for the development of highly antibacterial nanoparticles with essential oils. The encapsulation of these agents usually enhances its bacterial activity [34]. Ivanova et al. [35] prepared zein nanocapsules containing an antimicrobial essential oil, aminocellulose and a *S. aureus*-targeting antibody. It was found that this system attacked only the pathogen of interest. Zheng et al. [36] prepared carvacrol-loaded-zein nanoparticles with sodium caseinate and Bidyarany et al. [37] developed carvacrol-loaded-zein nanoparticles with rhamnolipids. In both cases the antimicrobial and antifungal activity of the loaded nanoparticles was higher than that of free carvacrol. Nanoparticles containing cationic surfactants are scarcely studied, and usually quaternary ammonium-based surfactants are used to prepare them. In this regard, the literature describes systems in which the activity of the antimicrobial surfactant increases when it is loaded in nanoparticles, while some in with the efficiency decreases have been also reported. Silica nanoparticles coated with didodecyldimethylammonium bromide showed lower MIC values against bacteria and fungi than soluble surfactant. It was found that their activity does not require the leaching of the surfactant from the surface of the nanoparticles [4,38]. Labena et al. [4] incorporated a new cationic surfactant into different nanoparticles. It was found that all the loaded nanoparticles presented higher antimicrobial activities than the synthesized surfactant alone. Mathiazzi and Carmona-Ribeiro (2020) studied the physical and antimicrobial properties of two quaternary ammonium-based surfactants, cetyl-trimethyl ammonium bromide and dioctadecyl dimethyl ammonium bromide in (methyl methacrylate) (PMMA) nanoparticles [39]. The systems were spherical and monodisperse but in this case the activity for the free surfactants was higher than that of the loaded-nanoparticles.

Regarding nanoparticles prepared with arginine-based surfactants, no references have been found in the literature. Nonetheless, the cationic lauroyl arginine ethyl ester (LAE) has been combined with polymers and zein to prepare antimicrobial films that allow to increase the stability of this cationic surfactant. Zein films with LAE were effective against both *L. monocytogenes* and *E. coli* and Chitosan films with LAE were active against Gram-positive and Gram-negative bacteria, yeasts, molds, and fungi [40].

Figure 12 shows the activity of these systems against some representative yeasts strains. Np-LAM-A exhibited very low MIC values against all microorganisms, it is noteworthy its high activity (MIC 4.45 μg/mL) against *C. auris*, a novel *Candida* species that has rapidly spread and shows resistance to antifungal drugs. The encapsulation of LAM in zein nanoparticles using the A method improves the activity of this surfactant against the tested strains (Figure 12). It is noticeable the low MIC values obtained for the NpLAM prepared by the method A; these aggregates are active against all Candida strains tested at MIC < 9 μg/mL (Figure 12). However, nanoparticles prepared using the B method had equal or lower activity than free LAM (Figure 12).

PNHC_12_ was also very potent, with MIC values < 9 µg/mL for all yeasts tested. The nanoparticles maintained the same pattern as in the antibacterial assay. While the preparation by method A removed the activity of the surfactant, similarly to that found for the bacteria, method-B maintained it in the same level. This result reinforces the different arrangement of the surfactant within zein molecular structure (Figure 10) according to the preparation method used and the extensive binding found (Figure 7). As a result, the nanoparticle microstructure will have the surfactant molecule and its active sites exposed differently. Additionally, the different size observed could affect the antifungal activity.

PANHC_12_ and C_12_PAM had similar activity against the yeasts. Their solutions and nanoparticles presented the same potency over the strains tested, indicating that the binding to zein did not hinder any effect over its pharmacological activity. It should be noted that these two surfactants as well as their nanoparticle formulations exhibited antifungal activity against all candida tested at MIC values around 8–36 μg/mL (Figure 12). This means that these nanoparticles can be also considered good antifungal systems.

Considering the MIC values obtained, it can be observed that in general these nanoparticles show a greater activity against yeasts than bacteria. It was observed that NpLAM prepared by method A was far more effective than LAM solution over all yeasts tested and the other surfactants retained good antifungal activity when incorporated in nanoparticles. For bacteria, a discrete attenuation in the activity of these amino acid based-surfactants was found when nanoencapsulated in zein nanoparticles, but, in general, these aggregates still maintain a very good antibacterial activity.

Moreover, blank zein nanoparticles (BZNp) did not show evident antimicrobial activity against the bacterial and yeast strains tested under the same conditions.

The obtained results indicate that the N-acyl derivatives, LAM and C_12_PAM, are two interesting systems to prepare eco-friendly antimicrobials nanoparticles given that these two cationic surfactants as well as zein are biodegradable and renewable materials. These aggregates can simplify the transport mode and also improve the stability of these cationic surfactants.

### 3.6. Hemolytic Activity

**Figure 13:**
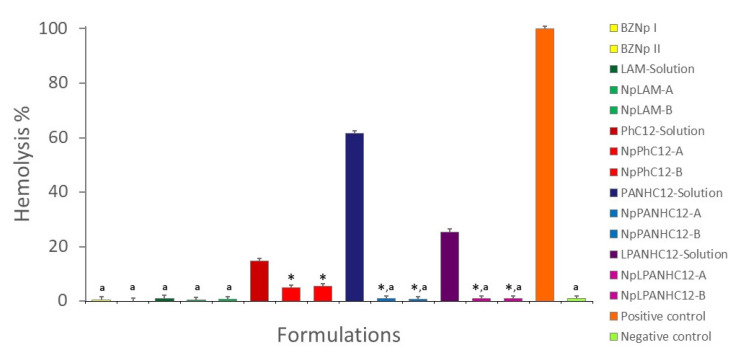
Hemolytic activity of the surfactants´ solutions (35.6 µg/mL) and loaded-zein nanoparticles prepared by method A and B. The results are expressed as percentage using the positive control as reference. Negative control was PBS 7.4 solution and positive control was ultrapure water. * Significatively reduced the hemolytic activity compared to the respective solution (*p* < 0.05). ^a^ Equal to the negative control (*p* > 0.05). Data submitted in pairs to one-way ANOVA with post hoc Tukey test when *p* < 0.05.

Selective toxicity for microorganisms is critical to the development of new antimicrobial formulations. The hemolytic activity of the surfactants’ nanoparticles and solutions was evaluated in vitro over erythrocytes cellular membranes (Figure 13). The tested concentration for all systems was fixed at 36.5 μg/mL, the same used in the nanoparticles.

Blank zein nanoparticles were found hemocompatible and did not produce any evident hemolysis. At the tested concentration, LAM solution was also non-hemolytic and the same behavior was observed for the LAM-Nanoparticles. Those formulations were found unhazardous to the erythrocytes in the same level as the negative control (*p* > 0.05).

PNHC_12_, C_12_PAM and most remarkably PANHC_12_ solutions exhibited different levels of hemolysis: PNHC_12_ 14.65%, C_12_PAM 25.41% and PANHC_12_ 62.7% (Figure 13). Remarkably, the encapsulation of the surfactants in zein nanoparticles reduced drastically hemolytic activity. The nanoencapsulation of PHNC_12_ reduced the hemolysis to 5.5%, while the hemolysis found for C_12_PAM and PANHC_12_ nanoparticles was below 1% (Figure 13), also equal to the negative control group (*p* > 0.05). Among these, no difference was found between methods A and B. The results indicate that the arginine-based surfactants, LAM, C_12_PAM and PANHC_12_, were not toxic at the concentrations effective against bacteria and yeast.

The reduction on the cytotoxicity for these systems can be attributed to the interactions found between the hydrophobic residues of zein and the alkyl chains of the cationic surfactants, which would make the amphiphile more internalized or immobilized. This performance can be also related to the size of the aggregates. In aqueous solutions, it is expected that these monocatenary cationic surfactants contains spherical micelles, thus when these compounds are loaded in zein nanoparticles the size of the aggregates increases a lot and the haemolysis diminish substantially. These results agree with that obtained by combining arginine-Gemini surfactants with cholesterol and dilauroyl phosphatidyl choline (DLPC) [29]. The combination of rhamnolipids and chitosan in nanoparticles also decreased their cytotoxicity while improved their antimicrobial activity [41]. Luis et al. also observed that zein nanoparticles had a protective effect against toxicity of antimicrobial essential oils. The LC_50_ of the encapsulated agent in zein nanoparticles was 2-fold the LC_50_ values of emulsions containing these active agents [42].

### 3.7. Limitations of the Present Study

In view of the results presented, it is notorious the potential of the nanoparticles of zein containing the arginine-phenylalanine surfactants in order to improve their biomedical applicability. Nonetheless, this work explores in vitro methodology, which is a first step in drug development. The results remark the adequacy, stability and chemical mechanisms of interactions between the surfactants and nanocarrier. Finally, the antimicrobial activity was accessed together with the haemolytic activity, demonstrating the higher selectivity of the surfactants when loaded to the nanoparticles. Nonetheless, to pursue clinical application, further in vitro experiments, for instance using different cell lines and exploring the action mechanisms over microbial biofilms together with in vivo assays of pharmacological activity and acute and or/chronic toxicity are needed.

## 4. Conclusions

Amino acid based-surfactants were loaded on zein nanoparticles, using the nanoprecipitation method. The nanoparticles were successfully prepared using two different approaches: A—solubilized together with zein before the nanoparticles’ formation and B—loaded on pre-formed zein nanoparticles. The preparation method used did not condition substantially the stability of the formulations. The nanoencapsulation of the surfactants allowed obtaining nanoparticles with suitable physicochemical and functional properties and different morphological characteristics. They were found stable in different storage conditions, with a long shelf-life (over 365 days).

In general, these nanoparticles show a greater activity against yeasts than bacteria. It was observed than NpLAM_A prepared by method A was far more effective than LAM solution over all yeasts tested and inhibited all the bacteria tested, while the other surfactants retained their excellent antifungal activity when incorporated in nanoparticles. For bacteria, a discrete reduction in the activity of these amino acid based-surfactants was found when nanoencapsulated in the zein nanoparticles, but, in general, these aggregates still maintain a very potent antibacterial activity.

The nanoencapsulation reduced extensively the hemolytic activity of all phenylalanine-based surfactants, improving their selectivity over microbial cell membranes. Those results agree with the NMR and docking findings, indicating that zein interacts with the surfactants by the aromatic and aliphatic chain and as a result interferes in the surfactant-lipid interaction needed for the microbial and cellular interactions. As a result, the cationic charges are freely available to attack and destroy the bacteria and fungi, while the aliphatic chain and aromatic rings are not free to disrupt the cellular membranes.

## Data Availability

Not applicable.

## Supplementary Materials

The following supporting information can be downloaded at: https://www.mdpi.com/article/10.3390/nano13010200/s1, Table S1: Results of interaction details and docking score in (kJ/mol) of LAM ligand complexed in different binding sites on the surfaces on the zein structures (Q9SYT3_MAIZE), Table S2: Results of interaction details and docking score in (kJ/mol) of PNHC_12_ ligand complexed in different binding sites on the surfaces on the zein structures (Q9SYT3_MAIZE), Table S3: Results of interaction details and docking score in (kJ/mol) of C_12_PAM ligand complexed in different binding sites on the surfaces on the zein structures (Q9SYT3_MAIZE), Table S4: Results of interaction details and docking score in (kJ/mol) of PANHC12 ligand complexed in different binding sites on the surfaces on the zein structures (Q9SYT3_MAIZE). Figure S1: Canonical forms of guanidine group.
