# Supplementary material for: Zein Nanoparticles Containing Arginine-Phenylalanine-Based Surfactants: Stability, Antimicrobial and Hemolytic Activity
Source: Nanomaterials (Basel). 2023 Jan 2;13(1):200. doi: 10.3390/nano13010200 (PMC9824401; doi:10.3390/nano13010200)
Supplement: Supplementary file 1 [file nanomaterials-13-00200-s001.zip › nanomaterials-2102771-supplementary.pdf]

nanomaterials

MDPI

Supplementary Materials

Zein Nanoparticles Containing Arginine-Phenylalanine-Based Surfactants: Stability, Antimicrobial and Hemolytic Activity

Lourdes Perez [1,*], Zakaria Hafidi [1], Aurora Pinazo [1], Maria Teresa García [1], Manuel Martín-Pastor [2] and Francisco Fábio Oliveira de Sousa [3,*]

[1] Department of Surfactants and Nanobiotechnology, Institute for Advanced Chemistry of Catalonia (IQAC-CSIC), 08034 Barcelona, Spain; zakariahafidi21@gmail.com (Z.H.); aurora.pinazo@iqac.csic.es (A.P.); teresa.garcia@iqac.csic.es (M.T.G.)
[2] Unidad de Resonancia Magnética, Área de Infraestructuras de Investigación, Universidad de Santiago de Compostela, Santiago de Compostela, 15782 A Coruña, Spain; manuel.martin@usc.es
[3] School of Pharmacy, Department of Biological & Health Sciences, Federal University of Amapa, Macapa 68903-419, Brazil
* Correspondence: lourdes.perez@iqac.csic.es (L.P.); fabio@unifap.br (F.F.O.d.S.)

Table S1. Results of interaction details and docking score in (kJ/mol) of LAM ligand complexed in different binding sites on the surfaces on the zein structures (Q9SYT3_MAIZE).

Docking Score	Pocket	Ligand	Receptor Pocket	Interactions type	Interactions category	Distance (Å)
−7.3	1	**H(N+H2 Guanidine)**	**PRO170**	**Hydrogen Bond**	**Conventional Hydrogen Bond**	2.97432
		H(N+H2 Guanidine)	PRO170	Hydrogen Bond	Conventional Hydrogen Bond	2.79542
		C(CH)	SER160	Hydrogen Bond	Carbon Hydrogen Bond	3.04949
		C(OCH3)	GLN205	Hydrogen Bond	Carbon Hydrogen Bond	3.23101
−6.9	2	C(CH3)	ALA184	Hydrophobic	Alkyl	3.65242
−7.3	3	H(N+H2 Guanidine)	PRO170	Hydrogen Bond	Conventional Hydrogen Bond	2.97888
		H(N+H2 Guanidine)	PRO170	Hydrogen Bond	Conventional Hydrogen Bond	2.77512
		C(CH)	SER160	Hydrogen Bond	Carbon Hydrogen Bond	3.08053
		C(OCH3)	GLN205	Hydrogen Bond	Carbon Hydrogen Bond	3.23649
−7.8	4	O(C=O)	HIS80	Hydrogen Bond	Carbon Hydrogen Bond	3.17117
		C(CH3)	LEU110	Hydrophobic	Alkyl	4.41005
		C(CH3)	LEU114	Hydrophobic	Alkyl	4.79395
−7.3	5	H(N+H2 Guanidine)	PRO170	Hydrogen Bond	Conventional Hydrogen Bond	3.0087

		H(N+H2 Guanidine)	PRO170	Hydrogen Bond	Conventional Hydrogen Bond	2.83475
		C(CH)	SER160	Hydrogen Bond	Carbon Hydrogen Bond	3.06283
		C(OCH3)	GLN205	Hydrogen Bond	Carbon Hydrogen Bond	3.25599
**−7.8**	6	C(OCH3)	GLN90	Hydrogen Bond	Carbon Hydrogen Bond	3.61402
		C(CH3)	ILE83	Hydrophobic	Alkyl	4.7638
**−6.1**	7	H(NH)	ASN20	Hydrogen Bond	Conventional Hydrogen Bond	1.83877
		H(N+H2 Guanidine)	THR19	Hydrogen Bond	Conventional Hydrogen Bond	2.49037
		C(CH3)	LEU12	Hydrophobic	Alkyl	5.30679
		C(CH3)	PHE15	Hydrophobic	Pi-Alkyl	4.35607

Table S2. Results of interaction details and docking score in (kJ/mol) of $PNHC_{12}$ ligand complexed in different binding sites on the surfaces on the zein structures (Q9SYT3_MAIZE).

Docking Score	Pocket	Ligand	Receptor Pocket	Interactions type	Interactions category	Distance (Å)
**−6.4**	1	**Ring**	**LEU177**	**Hydrophobic**	**Pi-Sigma**	3.95701
		C(CH3)	ALA123	Hydrophobic	Alkyl	3.69688
		C(CH3)	ALA169	Hydrophobic	Alkyl	4.48497
		C(CH3)	LEU164	Hydrophobic	Alkyl	4.52804
**−6.2**	2	H(N+H3)	SER162	Hydrogen Bond	Conventional Hydrogen Bond	1.96179
		H(N+H3)	LEU159	Hydrogen Bond	Conventional Hydrogen Bond	2.54609
		H(N+H3)	LEU159	Hydrogen Bond	Conventional Hydrogen Bond	2.99719
		O(C=O)	SER160	Hydrogen Bond	Carbon Hydrogen Bond	3.33147
		C(CH3)	ALA126	Hydrophobic	Alkyl	3.69262
		C(CH3)	ALA127	Hydrophobic	Alkyl	4.11668
		C(CH3)	LEU164	Hydrophobic	Alkyl	4.00228
		Ring	ALA165	Hydrophobic	Pi-Alkyl	5.04013
		Ring	PRO170	Hydrophobic	Pi-Alkyl	4.52456
**−6.1**	3	Ring	LEU177	Hydrophobic	Pi-Sigma	3.78559
		Ring	LEU177	Hydrophobic	Pi-Sigma	3.79241
		C(CH3)	ALA127	Hydrophobic	Alkyl	4.0609
		C(CH3)	LEU164	Hydrophobic	Alkyl	3.96071
		C(CH3)	LEU173	Hydrophobic	Alkyl	4.61468
**−6.1**	4	Ring	LEU177	Hydrophobic	Pi-Sigma	3.96935
		C(CH3)	ALA169	Hydrophobic	Alkyl	4.14848
		C(CH3)	LEU164	Hydrophobic	Alkyl	4.00945
**−6.1**	5	H(N+H3)	SER160	Hydrogen Bond	Conventional Hydrogen Bond	2.57972
		H(NH)	SER160	Hydrogen Bond	Conventional Hydrogen Bond	2.85029
		Ring	LEU177	Hydrophobic	Pi-Sigma	3.93429
		Ring	LEU177	Hydrophobic	Pi-Sigma	3.72676
		C(CH3)	ALA127	Hydrophobic	Alkyl	3.92817
		C(CH3)	ALA169	Hydrophobic	Alkyl	4.40679

		C(CH3)	PRO170	Hydrophobic	Alkyl	4.69182
		C(CH3)	LEU173	Hydrophobic	Alkyl	4.20458
**−5.3**	6	Ring	TYR128	Hydrophobic	Pi-Pi Stacked	3.73648
		C(CH3)	ILE83	Hydrophobic	Alkyl	4.01167
		Ring	ALA87	Hydrophobic	Pi-Alkyl	4.57961
**−4.3**	7	H(N+H3)	ALA54	Hydrogen Bond	Conventional Hydrogen Bond	2.17001
		H(N+H3)	GLN58	Hydrogen Bond	Conventional Hydrogen Bond	1.85122
		C(CH2)	ASN20	Hydrogen Bond	Carbon Hydrogen Bond	3.4593
		Ring	LEU57	Hydrophobic	Amide-Pi Stacked	4.68191
		C(CH3)	LEU57	Hydrophobic	Alkyl	3.99966
		Ring	LEU61	Hydrophobic	Pi-Alkyl	4.94572

Table S3. Results of interaction details and docking score in (kJ/mol) of C$_{12}$PAM ligand complexed in different binding sites on the surfaces on the zein structures (Q9SYT3_MAIZE).

Docking Score	Pocket	Ligand	Receptor Pocket	Interactions type	Interactions category	Distance (Å)
**−7.2**	**1**	**O(C=O)**	**ASN124**	**Hydrogen Bond**	**Conventional Hydrogen Bond**	2.43402
		H(NH)	ASN124	Hydrogen Bond	Conventional Hydrogen Bond	2.48521
		H(NH2 Guanidine)	ASN124	Hydrogen Bond	Conventional Hydrogen Bond	2.25149
		H(N+H2 Guanidine)	ALA169	Hydrogen Bond	Conventional Hydrogen Bond	1.82756
		Ring	LEU120	Hydrophobic	Pi-Alkyl	4.99389
**−7.9**	2	C(CH$_3$)	LEU164	Hydrophobic	Alkyl	4.66388
		C(CH3)	PRO170	Hydrophobic	Alkyl	4.54668
		C(CH3)	LEU173	Hydrophobic	Alkyl	4.29757
		Ring	LEU159	Hydrophobic	Pi-Alkyl	5.28949
**−7.9**	3	C(CH3)	LEU177	Hydrophobic	Alkyl	5.2232
		C(CH3)	PHE203	Hydrophobic	Pi-Alkyl	4.47273
		Ring	ALA165	Hydrophobic	Pi-Alkyl	3.86353
**−7.4**	4	Ring	ALA121	Hydrophobic	Pi-Sigma	3.6446
		Ring	LEU114	Hydrophobic	Pi-Alkyl	4.73884
**−8.2**	5	O(C=O)	GLN205	Hydrogen Bond	Conventional Hydrogen Bond	2.42986
		H(NH2 Guanidine)	SER160	Hydrogen Bond	Conventional Hydrogen Bond	2.87177
		O(C=O)	SER161	Hydrogen Bond	Carbon Hydrogen Bond	3.457
		Ring	PRO202	Hydrophobic	Pi-Alkyl	4.95903
**−7.4**	6	O(C=O)	GLN132	Hydrogen Bond	Conventional Hydrogen Bond	2.23862
		C(CH$_2$ Hydrophobic alkyl)	TYR109	Hydrophobic	Pi-Sigma	3.67581
		Ring	ALA87	Hydrophobic	Pi-Alkyl	4.1366
**−7.9**	7	H(NH2 Guanidine)	THR19	Hydrogen Bond	Conventional Hydrogen Bond	2.92367
		H(NH2 Guanidine)	THR19	Hydrogen Bond	Conventional Hydrogen Bond	2.74286
		H(NH2 Guanidine)	ASN20	Hydrogen Bond	Conventional Hydrogen Bond	2.48226
		Ring	LEU57	Hydrophobic	Pi-Sigma	3.91688

		Ring	LEU61	Hydrophobic	Pi-Alkyl	4.81839

Table S4. Results of interaction details and docking score in (kJ/mol) of PANHC12 ligand complexed in different binding sites on the surfaces on the zein structures (Q9SYT3_MAIZE).

Docking Score	Pocket	Ligand	Receptor Pocket	Interactions type	Interactions category	Distance (Å)
**−9**	**1**	**O(C=O)**	**THR207**	**Hydrogen Bond**	**Conventional Hydrogen Bond**	1.83822
		O(C=O)	THR207	Hydrogen Bond	Conventional Hydrogen Bond	1.7741
		H(N+H3)	GLN205	Hydrogen Bond	Conventional Hydrogen Bond	2.25351
**−7.7**	2	H(NH2 Guanidine)	SER162	Hydrogen Bond	Conventional Hydrogen Bond	2.18277
		H(NH2 Guanidine)	SER161	Hydrogen Bond	Conventional Hydrogen Bond	2.81668
		H(NH)	LEU158	Hydrogen Bond	Conventional Hydrogen Bond	2.27105
		H(N+H3)	GLN133	Hydrogen Bond	Conventional Hydrogen Bond	1.86787
		Ring	PHE137	Hydrophobic	Pi-Pi T-shaped	5.39863
		C(CH3)	PRO119	Hydrophobic	Alkyl	5.44349
**−9.1**	3	O(C=O)	THR207	Hydrogen Bond	Conventional Hydrogen Bond	1.82891
		O(C=O)	THR207	Hydrogen Bond	Conventional Hydrogen Bond	2.15488
		H(N+H3)	GLN205	Hydrogen Bond	Conventional Hydrogen Bond	2.20747
**−9.1**	4	O(C=O)	HIS80	Hydrogen Bond	Conventional Hydrogen Bond	2.40394
		H(N+H3)	ASN124	Hydrogen Bond	Conventional Hydrogen Bond	2.61522
		Ring	ALA87	Hydrophobic	Pi-Sigma	3.99029
**−6.8**	5	H(N+H3)	LEU225	Hydrogen Bond	Conventional Hydrogen Bond	2.03435
		H(CH)	GLU226	Hydrogen Bond	Carbon Hydrogen Bond	3.47424
		Ring	LEU236	Hydrophobic	Pi-Sigma	3.71367
		Ring	LEU222	Hydrophobic	Pi-Alkyl	5.47085
**−9.8**	6	Ring	TYR128	Hydrophobic	Pi-Pi Stacked	3.85476
		Ring	ALA87	Hydrophobic	Pi-Alkyl	4.53503
		Ring	VAL125	Hydrophobic	Pi-Alkyl	5.28344
**−7.5**	7	O(C=O)	PHE22	Hydrogen Bond	Conventional Hydrogen Bond	2.16811
		C(CH3)	ALA253	Hydrophobic	Alkyl	3.55668
		Ring	ALA54	Hydrophobic	Pi-Alkyl	5.25214
		Ring	LEU57	Hydrophobic	Pi-Alkyl	4.33549

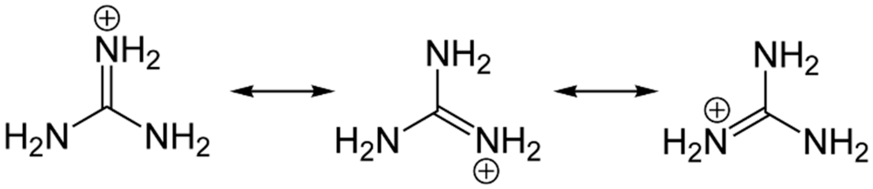

Figure S1. Canonical forms of guanidine group.
